# Continuous subcutaneous octreotide infusion in congenital hyperinsulinism: practical application and insights in infancy and early childhood

**DOI:** 10.1007/s40200-025-01709-2

**Published:** 2025-10-11

**Authors:** Mercedes J. Burnside, Adam Stafford-Bell, Rachel Romans, Matthew Moller, Catherine S. Choong, Mary B. Abraham

**Affiliations:** 1 Department of Endocrinology and Diabetes, Perth Children’s Hospital, Nedlands, Perth, WA Australia; 2 Medical School, The University of Western Australia, Perth, WA Australia; 3 Department of Pharmacy, Perth Children’s Hospital, Perth, WA Australia; 4 Centre for Child Health Research, The University of Western Australia, Perth, WA Australia

**Keywords:** Congenital hyperinsulinism, Octreotide, Pump, Continuous infusion

## Abstract

**Purpose:**

Octreotide is second-line treatment for patients with congenital hyperinsulinism (CHI) unresponsive to diazoxide. Short-acting octreotide is administered via subcutaneous (SC) injection or continuous infusion using a pump, but limited data exist on pump therapy. We present two cases of CHI managed with an octreotide infusion via pump with or without concurrent continuous glucose monitoring (CGM), providing practical guidance.

**Case presentation:**

Octreotide 500mcg/1ml ampoule was used. The starting dose was 5mcg/kg/day, with titration to optimize glycaemia, and delivered as a continuous basal rate using a pump. CGM was used by Patient 2.

Patient 1: CHI due to a paternal *ABCC8* mutation was detected at 16 weeks of age, with diffuse uptake on 18F-DOPA PET. Unresponsive to diazoxide, SC octreotide injections commenced, transitioning to an infusion (13mcg/kg/day) with good response, and complete cessation of therapy at four years.

Patient 2: Neonate with CHI due to Beckwith Wiedemann Syndrome developed side-effects from diazoxide and was commenced on SC octreotide injections. Effective at 30mcg/kg/day, he transitioned to an infusion at 4.5 months followed by long-acting octreotide at 12 months.

Both patients tolerated the infusion with no adverse effects.

**Conclusion:**

This report highlights the practical use of octreotide infusion via pump. When combined with CGM, it allows for tailored dosing to meet individual needs while enhancing patient convenience.

## Introduction

Congenital hyperinsulinism (CHI) is the most common cause of persistent hypoglycaemia in infants [1], presenting in both syndromic and isolated forms, with diffuse or focal histological variants [2]. CHI is characterized by dysregulated insulin secretion from pancreatic beta cells, most often due to inactivating mutations in the ATP-sensitive potassium (K_ATP_) channel genes (*ABCC8* and *KCNJ11*) located on chromosome 11p15.1 [3]. Prompt and effective treatment is crucial to support optimal neurodevelopment.

In diffuse CHI, medical management is preferred, with diazoxide serving as the first-line therapy [4]. However, cases due to mutations in K_ATP_ channel genes are often unresponsive to diazoxide. In such cases, octreotide, a somatostatin analogue, is commonly used as a second-line therapy [4–6]. Somatostatin, naturally secreted by pancreatic delta cells in response to glucose, amino acids, and glucagon-like peptide-1, binds to receptors on pancreatic beta cells. This binding inhibits voltage-gated calcium channels and K_ATP_ channels, reducing cyclic adenosine monophosphate levels and suppressing insulin secretion [7]. While the clinical use of somatostatin is limited by its short half-life of less than three minutes, octreotide, a synthetic analogue, offers a more favourable pharmacokinetic profile.

Both short-acting (SA) and long-acting (LA) octreotide preparations are used in the management of CHI. LA octreotide, administered monthly via intramuscular injection, provides a convenient alternative; however, there is lack of appropriate dosing and safety data in early infancy [8]. Therefore, our centre uses SA octreotide during the neonatal period and infancy until patients can be transitioned to LA octreotide.

SA octreotide has a recommended initial dose of 5mcg/kg/day, with a maximum of 35mcg/kg/day [5, 9]. It has a half-life of 90 to 120 min and exerts a pharmacodynamic effect lasting eight to 12 h [7]. Consequently, it must be administered either as subcutaneous (SC) multiple daily injections every six to eight hours or through continuous subcutaneous infusion via a pump. Both administration methods have been shown to be effective, safe, and well-tolerated [10], and clinical guidelines endorse their use as second-line treatment for CHI [4, 11].

Continuous glucose monitoring (CGM) provides insight into real-time glucose levels, allowing for adjustments to octreotide infusion therapy. Despite this advancement, there remains a lack of detailed guidance on the practical aspects of pump therapy and dose titration [12]. In this report, we present two cases of CHI successfully managed with continuous octreotide infusion, focusing on the individualized management of hypoglycaemia through tailored therapy.

## Case presentation

### Methods

In both infants, hyperinsulinism was identified as the cause of hypoglycaemia, diagnosed by detectable insulin levels during episodes of hypoglycaemia (< 2.8mmol/L) coupled with hypoketonemia (β-hydroxybutyrate < 1.8 mmol/L) [4]. Patient 1 was unresponsive to diazoxide, experiencing persistent hypoglycaemia despite five days of maximal treatment (20mg/kg/day) and Patient 2 developed significant side effects from diazoxide (15mg/kg/day). Patients were initially treated with SC octreotide injections, starting at a dose of 5mcg/kg/day, with adjustments made to maintain glucose levels above 3.5mmol/L. Once a stable dose was achieved and responsiveness to octreotide confirmed, transition to a continuous infusion occurred.

Octreotide was administered from 500mcg/1ml ampoules and delivered continuously via an insulin pump (pump rate calculation shown in Table 1). Glucose-enriched feeds were provided as needed to support therapy. Pumps were procured through the endocrine department, and CGM has been available for CHI through the national scheme since 2019. CGM (Dexcom G6) was offered to both families. Patient 2 utilized CGM to fine-tune both dietary and medical therapy based on real-time glucose data while Patient 1 elected to continue with self-monitoring of blood glucose levels using a glucometer.

**Table 1:** Octreotide calculation for delivery via an insulin pump

Preparation:
Short-acting octreotide 500 mcg/mL from vials or ampoules
Starting Dose:
Use the patient’s current total daily subcutaneous (SC) octreotide dose (TDD), or commence at 5 mcg/kg/day
Pump Consideration:
Insulin pumps display rate as units per hour; thus, conversion is required Insulin pumps are calibrated for insulin at 100 units/mL (i.e. 1 unit/0.01 mL), where: • 1 unit/hour = 0.01 mL/hour • At a dilution of 500 mcg/mL, this equals 5 mcg octreotide/hour
Calculation:
1. **Octreotide rate in mcg/hour** = TDD ÷ 24 2. **Octreotide rate in units/hour** = (mcg/hour) ÷ 5 3. Alternatively, **units/hour** = TDD ÷ 120 → Round the result to an appropriate pump-deliverable rate
Worked Example:
**Patient Dose:** 80 mcg SC every 8 h = 240 mcg/day 1. mcg/hour = 240 ÷ 24 = 10 mcg/hour 2. units/hour = 10 ÷ 5 = **2 units/hour** to program on the pump

Families received comprehensive training in pump management during their inpatient stay. This training covered the procedures for changing the pump cannula and tubing, as well as replacing octreotide every three days. They were also instructed on the risks of pump disconnection and malfunction. All necessary pump supplies were provided by our department, and octreotide was dispensed by the hospital pharmacy. An action plan for pump-failure and hypoglycaemia management with emergency contact details was provided to the families.

Following discharge, regular follow-up appointments were scheduled to monitor glycaemia through self-monitoring of blood glucose and/or CGM, and to assess for potential adverse effects. Octreotide dose adjustments were made based on glucose levels, aiming for 3.5–5.5mmol/L, avoiding hypoglycaemia (< 3.5mmol/L), and achieving age-appropriate fasting duration. Routine follow-up evaluations included growth assessments every three months, and every six months, gall bladder ultrasounds to screen for cholelithiasis and laboratory tests to monitor liver enzymes, growth factors, and thyroid function.

Written informed consent was obtained from a parent of each patient for publication of this case series according to the Human Research Ethics Committee procedures of the hospital.

### Cases

Patient 1: A female infant, born at term and weighing 4.2kg, experienced a hypoglycaemic seizure at four months of age due to hyperinsulinism. Although genetic testing revealed a paternally inherited *ABCC8* missense mutation, c.107A > G, suggestive of a focal lesion, an 18F-DOPA PET CT scan demonstrated diffuse pancreatic uptake. The infant did not respond to diazoxide, so octreotide was administered SC every six hours with an adequate response. At five months of age, the regimen was switched to an octreotide infusion using a Medtronic MiniMed 640G pump, delivering 13mcg/kg/day at a constant rate over 24 h, which proved effective.

Glucose-enriched feeds were not required. She was maintained on a regular diet with attention to optimal carbohydrate intake. Regular self-monitoring of blood glucose before meals and bedtime demonstrated levels in target range. A pump malfunction, without serious hypoglycaemia, led to hospital admission and temporary transition to SC octreotide injections until a replacement pump was obtained. The family was offered the option to switch to LA octreotide after the child reached one year of age. However, they chose to continue with the continuous infusion, citing concerns about the discomfort of injections, as reported by other families in social media groups.

The dosage of octreotide gradually reduced over time as the child grew, eventually reaching less than 5mcg/kg/day. By the age of three, the infusion was needed only overnight, with pump disconnection during the day. By four years of age, therapy was completely discontinued, and no developmental concerns were reported. A fasting study supported resolution of hyperinsulinism; after a 16-h fast blood glucose was 2.7mmol/L, insulin 1mU/L, β-hydroxybutyrate 1.8mmol/L.

Patient 2: A male infant born at 37 weeks' gestation, weighing 4.3kg, was prenatally diagnosed with Beckwith-Wiedemann Syndrome (BWS) due to mosaic paternal uniparental disomy (*pUPD11p*). The infant developed persistent hypoglycaemia attributed to hyperinsulinism. Clinical features included macroglossia requiring CPAP for respiratory support and primary closure of exomphalos on day one. The maximal glucose infusion rate (GIR) was 19mg/kg/min. Diazoxide and hydrochlorothiazide improved glucose levels but led to fluid overload without pulmonary hypertension, and respiratory deterioration, necessitating cessation of diazoxide. An 18F-DOPA PET CT scan revealed diffuse uptake without focal abnormalities and massively parallel sequencing of monogenic hyperinsulinism genes was normal (inclusive of *ABCC8, KCNJ11, GLUD1, HNF4A, GCK, HADH, INSR, SLC16A1, TRMT10A, HNF1A, AKT2, CACNA1D*).

**Fig. 1:**
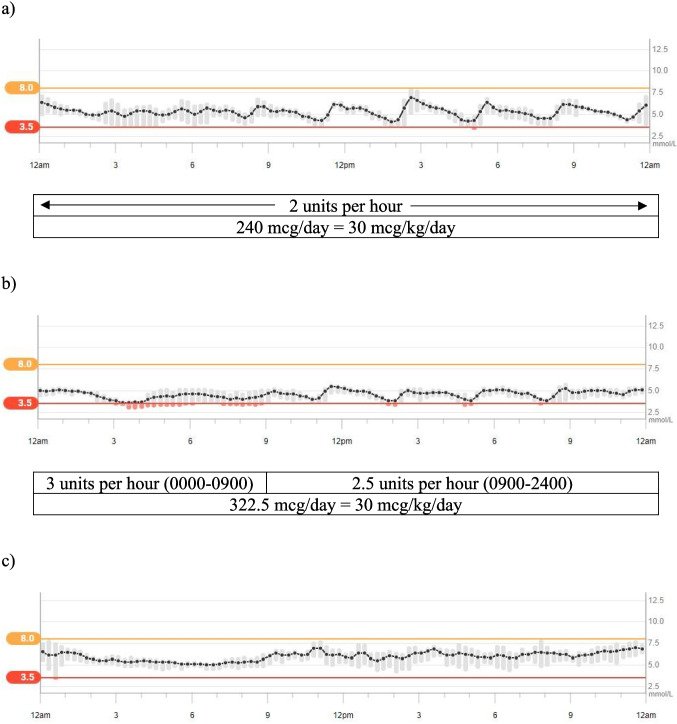
Continuous glucose monitoring data (averaged over a 7-day period) and octreotide infusion pump rates for Patient 2. **a**, 4.5 months of age, after initiation of octreotide continuous subcutaneous infusion. **b**, 10 months of age, before increasing pump rates between 0000–0900 h. **c**, 16 months of age, following transition to long-acting intramuscular octreotide

Octreotide was commenced at four months of age, administered SC every six hours, titrated to 30mcg/kg/day, effectively managing hypoglycaemia. Two weeks later, following resolution of fluid overload, CGM and an octreotide infusion (using a Medtronic MiniMed 770G pump) were initiated alongside three hourly nasogastric tube feeds of term formula with added glucose (2% glucose polymer supplement), achieving 94% time in range (TIR; 3.5-8mmol/L) without hypoglycaemia < 3mmol/L (shown in Fig. 1. a). At ten months of age, a high time below range (TBR; < 3.5mmol/L) of 12% was observed (shown in Fig. 1. b). To address this, pump rates were adjusted to deliver more octreotide between 0000 and 0900 h, during the longer periods between feeds.

At 12 months of age, the patient successfully transitioned to LA octreotide. By 16 months, the infant was on full oral feeds without added calories or nocturnal feeding, maintaining a 100% TIR (shown in Fig. 1. c). The patient remains under paediatric follow-up for mild gross motor delay and tumour surveillance.

**Fig. 2:**
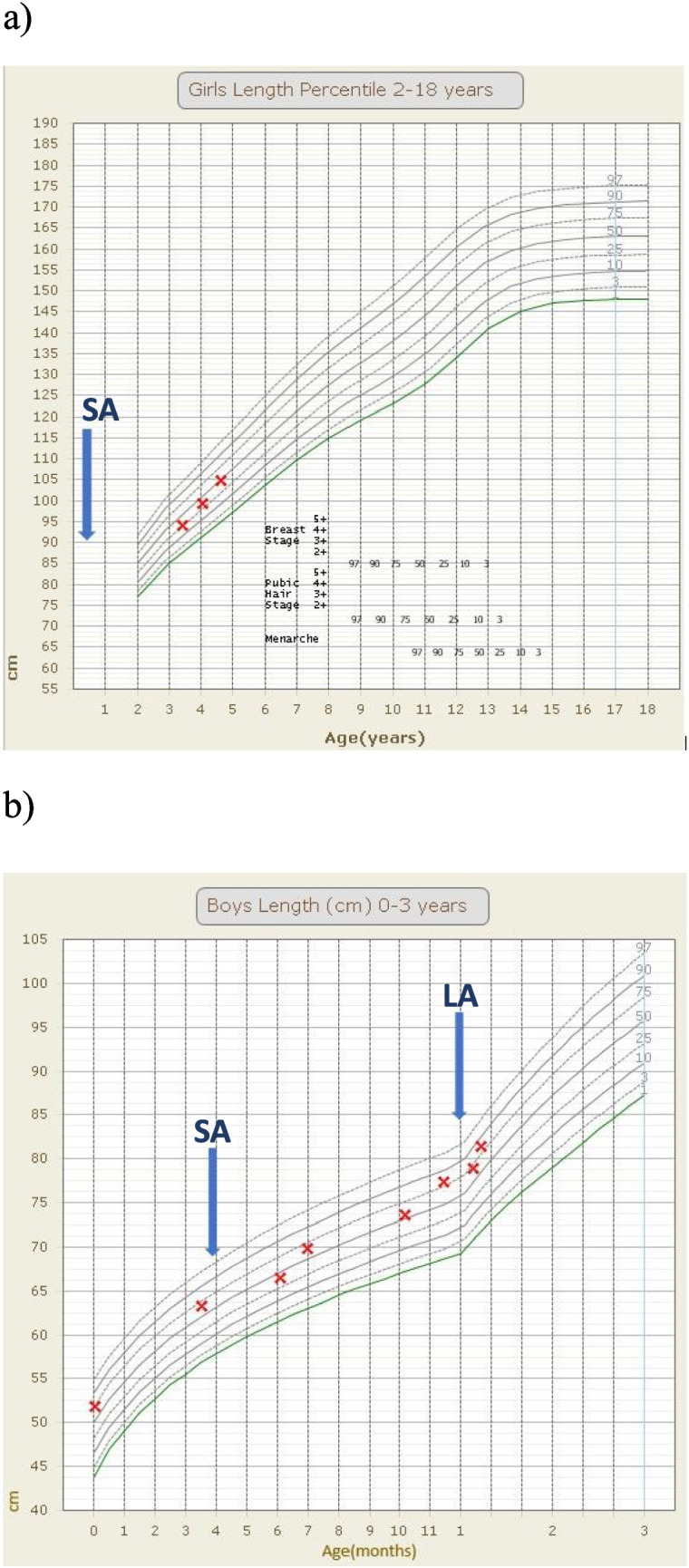
Linear growth charts with blue arrows indicating time of initiation of octreotide therapy (SA is short-acting and LA is long-acting). **a**, Patient 1, octreotide continuous subcutaneous infusion started aged four months. **b**, Patient 2, subcutaneous octreotide injections at four months and transitioned to a long-acting analogue at 12 months of age

Both patients tolerated octreotide infusions, with Patient 1 receiving treatment for the longest duration of 3.5 years. No adverse effects from octreotide infusion were observed in either patient, including normal thyroid hormone and liver enzyme levels, no cholelithiasis on ultrasonography, and appropriate linear growth (shown in Fig. 2).

## Discussion

These cases present an innovative approach to managing hyperinsulinism using a protocol that combines continuous octreotide infusion via pump with CGM or self-monitoring of blood glucose. By repurposing diabetes technologies, we enable precise therapy adjustments and enhanced glucose control. This approach offers a practical alternative to standard treatment, improving convenience and reducing the burden on patients and caregivers.

Octreotide administered via continuous infusion using a pump offers several advantages over SC injections. First, therapy can be precisely tailored by adjusting pump rates, as demonstrated in Patient 2, where a higher overnight basal rate was used to manage a vulnerable period with fewer feeds. Additionally, temporarily disconnecting the pump during the day when children eat regularly, as seen in Patient 1, allows further customization of therapy and is more suitable for childhood routines. This level of personalization aligns with findings from Tiberi et al., who utilized adjustable pump rates in an infant with CHI [12]. Second, continuous infusion reduces the need for multiple injections, likely minimizing distress to the child and caregivers and improving adherence. Third, studies suggest that octreotide dosing via infusion is typically lower than with multiple SC injections, potentially reducing the risk of side effects [5]; however, reduced dose via pump was not seen in our patients. Furthermore, in the Japanese SCORCH (subcutaneous octreotide for congenital hyperinsulinism) registry, parents expressed a preference for continuous infusion over multiple SC injections, reinforcing the acceptability of this approach [10]. Hence, utilizing a continuous infusion approach to refine therapy and reduce the number of injections is a valuable treatment strategy.

At our centre, octreotide is only available as 500mcg/ml, though other facilities may have more dilute preparations. Transition from multiple daily SC injections to pump therapy and home management require planning. The small doses required for SC injection necessitates aseptic dilution to achieve a deliverable volume, introducing potential for dosing errors if continued at home [13]. To mitigate this risk, children are initially managed with SC octreotide injections in an inpatient setting. If they respond to treatment, they are transitioned to an octreotide infusion via pump before discharge. Pump therapy entails a risk of device malfunction, as seen with Patient 1, where readmission was necessary for SC injections until a replacement pump was available. More recently, we have gained access to octreotide at a lower concentration (100mcg/ml), enabling patients to safely continue SC injections at home in the event of pump failure. Pump procurement can be another challenge, although this was mitigated by department procurement of pumps. Due to these challenges, patients are transitioned to LA octreotide at around 12 months of age.

LA octreotide preparations, administered as monthly injections, offer a convenient alternative to SA formulations and is an established long-term therapy for CHI [14]. However, there is a lack of efficacy and safety data along with limited dosing guidance in young infants, and hence SC octreotide can be considered in the interim before transitioning to LA octreotide. Concerns also exist regarding pain associated with deep intramuscular injections; to alleviate discomfort and anxiety in children, the use of topical anaesthesia may be considered [15].

Our two patients are from diverse sociodemographic backgrounds, and both reside in rural Western Australia. To support the families with successful pump therapy at home, we ensured a reliable supply of octreotide and pump consumables and provided them direct access to our endocrine liaison nurse, who fostered strong, supportive relationships. The patients received a personalized management plan for hypoglycaemia, which included the use of glucose powder and intramuscular glucagon for refractory cases, as well as guidance on addressing pump malfunctions, which entailed seeking hospital care for glucose stabilization. We collaborated with local paediatric healthcare teams and pharmacies to ensure access to octreotide and continued support. The patients were routinely reviewed in our dedicated hyperinsulinism clinic every three months. For Patient 2, CGM enabled remote monitoring of glucose levels facilitating timely adjustments to infusion rates between scheduled visits.

In both patients, continuous octreotide infusion led to improvements in glucose management. Furthermore, octreotide therapy facilitated a reduction in carbohydrate intake and normalized feeding schedules. The efficacy of SA octreotide administered via SC injections in treating CHI is well-established [6, 16], and small case series have specifically demonstrated that continuous octreotide infusions are both effective and safe [10, 16, 17]. Although continuous octreotide infusion demonstrates glycaemic benefits [5, 10, 15, 16], guidance on its practical application is limited, a gap that we have addressed in this report.

The effectiveness of octreotide may be constrained by tachyphylaxis [6], and important side effects include necrotizing enterocolitis, suppression of growth factors and thyroid hormones, liver enzyme abnormalities, and gallstones [18]. Most side effects are mild; the SCORCH data indicated no severe adverse effects over 695.4 patient-months [10]. A review by Welters et al. found that 31% of 355 patients experienced mild adverse events, primarily gastrointestinal symptoms [18]. Growth deceleration reportedly occurs in fewer than 5% of cases and is associated with higher doses [5, 18]. Similarly, doses exceeding 30mcg/kg/day have been linked to cholestatic jaundice and gallstones [18]. Therefore, it is essential to prioritize the lowest effective dose, achievable through CGM and precise infusion adjustments. In our patients, no adverse effects were observed. Both patients demonstrated normal growth, with no serious hypoglycaemic events related to pump disconnections or cannula occlusions.

## Conclusion

In conclusion, continuous subcutaneous octreotide infusion proved to be an effective and personalized treatment option for managing CHI in our two cases. It optimized glucose levels, reduced the need for surgical intervention, and normalized feeding schedules, while minimizing adverse effects. The use of CGM further enhanced the ability to fine-tune therapy, resulting in individualized care that was well-tolerated by patients and manageable for families. Our findings contribute to the limited practical guidance on the use of octreotide pump therapy and emphasize the importance of personalized dosing strategies in optimizing outcomes for patients with CHI.

## What is known

1. Octreotide is second-line treatment for patients with congenital hyperinsulinism unresponsive to diazoxide or experiencing side effects.
2. Short-acting octreotide is administered either as subcutaneous multiple daily injections or as a continuous subcutaneous infusion via a pump designed for insulin delivery.
3. Both administration methods have been shown to be effective and safe, and clinical guidelines endorse their use as second-line treatment. However, there is a lack of detailed guidance on the practical aspects of pump therapy.

## What is novel

1. This report contributes to the practical guidance on the use of octreotide pump therapy and dose titration to support healthcare professionals on this therapeutic approach.
2. We demonstrate how octreotide via a pump allows therapy to be precisely tailored by adjusting pump rates to optimize outcomes for patients.
3. The use of CGM further enhances the ability to fine-tune therapy, resulting in individualized care that is well-tolerated by patients and manageable for families.

## Data Availability

No datasets were generated or analysed during the current study.

## Declarations

### Study approval

Ethics approval was not required for this case report as per the Human Research Ethics Committee procedures of the hospital. This retrospective review of patient data did not require ethical approval in accordance with local guidelines.
